# Solid tumor size for prediction of recurrence in large and giant non-functioning pituitary adenomas

**DOI:** 10.1007/s10143-021-01662-7

**Published:** 2021-10-04

**Authors:** Ching-Chung Ko, Chin-Hong Chang, Tai-Yuan Chen, Sher-Wei Lim, Te-Chang Wu, Jeon-Hor Chen, Yu‐Ting Kuo

**Affiliations:** 1 Department of Medical Imaging, Chi-Mei Medical Center, Tainan, Taiwan; 2 Department of Health and Nutrition, Chia Nan University of Pharmacy and Science, Tainan, Taiwan; 3 Department of Neurosurgery, Chi Mei Medical Center, Tainan, Taiwan; 4 Graduate Institute of Medical Sciences, Chang Jung Christian University, Tainan, Taiwan; 5 Department of Neurosurgery, Chi-Mei Medical Center, Chiali, Tainan, Taiwan; 6 Department of Nursing, Min-Hwei College of Health Care Management, Tainan, Taiwan; 7 Department of Radiological Sciences, University of California, Irvine, CA USA; 8 Department of Radiology, E-DA Hospital, I-Shou University, Kaohsiung, Taiwan; 9 Department of Medical Imaging, Kaohsiung Medical University Hospital, Kaohsiung, Taiwan

**Keywords:** Solid tumor size, Pituitary macroadenoma, Recurrence, Non-functioning, MRI

## Abstract

A subset of large non-functioning pituitary adenomas (lNFPA) and giant non-functioning pituitary adenomas (gNFPA) undergoes early progression/recurrence (P/R) after surgery. This study revealed the clinical and image predictors of P/R in lNFPA and gNFPA, with emphasis on *solid* tumor size. This retrospective study investigated the preoperative MR imaging features for the prediction of P/R in lNFPA (> 3 cm) and gNFPA (> 4 cm). Only the patients with a complete preoperative brain MRI and undergone postoperative MRI follow-ups for more than 1 year were included. From November 2010 to December 2020, a total of 34 patients diagnosed with lNFPA and gNFPA were included (median follow-up time 47.6 months) in this study. A total of twenty-three (23/34, 67.6%) patients had P/R, and the median time to P/R is 25.2 months. Solid tumor diameter (STD), solid tumor volume (STV), and extent of resection are associated with P/R (*p* < 0.05). Multivariate analysis showed large STV is a risk factor for P/R (*p* < 0.05) with a hazard ratio of 30.79. The cutoff points of STD and STV for prediction of P/R are 26 mm and 7.6 cm^3^, with AUCs of 0.78 and 0.79 respectively. Kaplan–Meier analysis of tumor P/R trends showed that patients with larger STD and STV exhibited shorter progression-free survival (*p* < 0.05). For lNFPA and gNFPA, preoperative STD and STV are significant predictors of P/R. The results offer objective and valuable information for treatment planning in this subgroup.

**Supplementary Information:**

The online version contains supplementary material available at 10.1007/s10143-021-01662-7.

## Introduction

Pituitary adenomas (PA) constitute 10–25% of all intracranial neoplasms [12]. A subgroup of these tumors particularly challenging to manage are those that can be classified as large and giant PA [4, 8]. Although there is no consensus regarding the exact definition of tumor size, the largest tumor diameter of > 4 cm is considered giant, whereas > 3 cm is considered large [5, 8, 19, 30, 34, 38]. Large and giant PA that grow beyond the sellar are always difficult to manage surgically because of the surrounding important neurovascular structures and a greater risk of complications [4, 8, 35]. Large and giant PA comprise about 6–10% of all pituitary tumors [12, 44]. Most of them are clinically non-functioning pituitary adenomas (NFPA) and occur predominantly in males [12, 17, 44]. Visual field defects resulting from compression of optic chiasm are the most common preoperative symptoms followed by a headache. Partial or total hypopituitarism is observed in some patients due to tumor compression of the normal pituitary gland [17]. Although more than 90% of NFPA are diagnosed as benign adenomas according to 2017 WHO classification [27], 12–46% of them may undergo early progression/recurrence (P/R) after surgical resection [6, 7, 9, 13, 40]. Gross-total resection (GTR) by transsphenoidal approach (TSA) is the standard surgical treatment in the majority of NFPA; however, it is difficult to achieve in large NFPA (lNFPA) and giant NFPA (gNFPA) [36]. Therefore, a relatively high P/R rate due to postoperative residual tumor had been reported in this subgroup [6, 7, 39]. Although adjuvant radiotherapy (RT) is implemented in some institutions to prevent P/R in NFPA, 20–30% of patients may have irreversible hypopituitarism or other complications after treatment [17].

MRI findings such as cavernous sinus invasion, absence of apoplexy, postoperative residual tumor, diffusion restriction, and radiomics score have been reported as significant parameters related to P/R in NFPA [21, 28, 46]. However, some of them are qualitative, and others need to be analyzed on advanced MRI sequences. In oncologic imaging, Response Evaluation Criteria in Solid Tumors (RECIST), based on simple one-dimensional morphologic measurement of tumor diameter, is the gold standard for assessment of treatment response in solid tumors [10, 43]. However, a modified RECIST was developed based on the concept that a viable tumor should be defined as only intratumoral enhancing *solid* mass in some tumors [24, 26]. Although large tumor size is associated with lesser extent of tumor resection and more surgical complications in NFPA [2, 13, 28, 36, 41], the preoperative quantitative tumor size for the prediction of postoperative recurrence in NFPA was rarely mentioned. Further, no reports regarding the concept of simple measurements of *solid* tumor size for the prediction of clinical outcomes in NFPA have been published as of yet. This study evaluated the preoperative clinical and MR imaging characteristics for the prediction of P/R in lNFPA and gNFPA, with emphasis on solid tumor diameter (STD) and solid tumor volume (STV).

## Materials and methods

### Ethics statement

This study was approved by the Institutional Review Board (IRB no. 10902–009). Written consent was waived because the retrospective nature of this study does not influence the health care of the included patients. All patients’ medical records and imaging are anonymized and de-identified prior to analysis.

### Patient selection

The inclusion criteria of this study are patients diagnosed with large (> 3 cm) or giant (> 4 cm) NFPA by brain MRI and pathological confirmation, and with post-operative follow-up MRIs (at least 2 times) more than 1 year after treatment. Patients with clinical, biochemical, or histopathological evidence of hormone hypersecretion are excluded. Diagnosis of prolactinoma is considered unlikely if the prolactin levels are below 100 mg/L according to previous studies [2, 16], and a conclusion thereafter confirmed by immunocytochemical studies. Patients receiving postoperative adjuvant RT before P/R are also excluded. From September 2010 to December 2020, 292 patients are diagnosed with PA in our institution. Thirty-four patients (34/292, 11.6%) (21 men, 13 women, age 20–80 years; median age, 49.5 years) diagnosed with lNFPA and gNFPA are included in this study by the above-mentioned inclusion and exclusion criteria. Among them, thirty-two patients underwent surgery performed by TSA, and 2 patients received both TSA and craniotomy due to large tumor sizes. Fourteen (14/34, 41.2%) patients received repeated surgery due to tumor recurrence. The median follow-up duration for all patients is 47.6 months (range from 12 to 115 months). In 23 patients with P/R, the median time to P/R is 25.2 months (range from 6 to 67 months). Clinical and biochemical data are obtained from medical records.

### Extent of resection and progression/recurrence

The extent of resection (EOR) is determined by reviewing postoperative MRI by a neuroradiologist (C.C.K.) and a neurosurgeon (S.W.L.). According to published literatures [21, 45, 46], GTR is defined as a lesion with a residual tumor volume of less than 10% of its original size. In contrast, subtotal resection (STR) is defined as the presence of a residual tumor more than 10% of its original volume. For determining P/R in the included NFPA patients, pretreatment and postoperative MR images are evaluated by two experienced neuroradiologists (C.C.K. with 7 years of experience and T.Y.C. with 20 years of experience), both of whom are blinded to the clinical outcomes of the studied population. P/R is defined as tumor recurrence after GTR or enlargement of residual tumor after STR based on postoperative contrast-enhanced (CE) T1WI. According to published literatures [2, 21, 46], the threshold of P/R is defined as a more than 2-mm increase in size of residual tumor in at least one dimension when compared with postoperative MRI studies. Inter-observer reliability in the determination of P/R is obtained via a Cohen *k* value of 0.9. Judgment is made via consensus for equivocal cases. The preoperative MRI findings, including cavernous sinus invasion (Knosp classification) [20], extrasellar extension (Hardy’s classification) [15], compression of the optic chiasm and the third ventricle, hydrocephalus, and intratumoral apoplexy or cystic change are determined on coronal T2WI and CE T1WI.

### Imaging acquisition

Preoperative brain MRI images are acquired with a 1.5-T (Siemens, MAGNETOM Avanto) (*n* = 18), 1.5-T (GE Healthcare, Signa HDxt) (*n* = 10), or a 3-T (GE Healthcare, Discovery MR750) (*n* = 6) MRI scanner equipped with an 8-channel head coil in each machine. Scanning protocols include axial and sagittal spin-echo T1-weighted imaging (T1WI), axial and coronal fast spin-echo T2-weighted imaging (T2WI), axial fluid-attenuated inversion recovery, axial T2*-weighted gradient-recalled echo, and coronal and sagittal contrast-enhanced (CE) T1WI with fat saturation. Dynamic CE T1WI with a small field of view on the pituitary gland is also performed. For CE imaging, intravenous administration of 0.1 mmol/kg of body weight of gadobutrol or gadoterate meglumine is performed. Detailed imaging parameters in the MRI scanners are described in supplementary file 1.

### Measurement of tumor diameter and volume

**Fig. 1:**
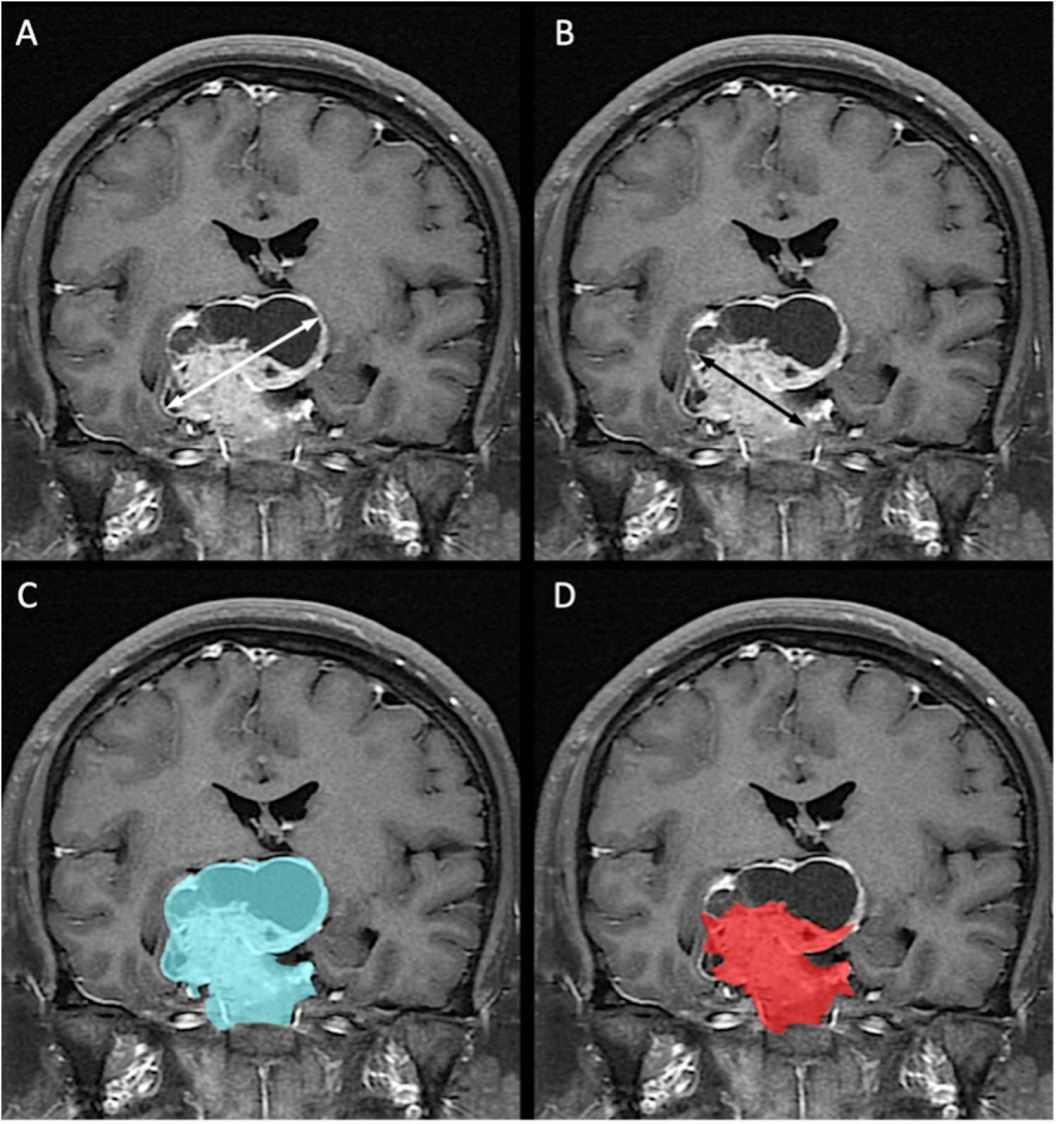
Example of measurements of preoperative solid tumor diameter (STD) and solid tumor volume (STV) on coronal contrast-enhanced (CE) T1WI in large nonfunctioning pituitary adenomas (lNFPA) and giant nonfunctioning pituitary adenomas (gNFPA). **A** Conventionally, both solid and cystic components were included in the measurement of maximal tumor diameter (MTD) (white arrow) (53 mm). **B** In contrast, STD was defined as a measurement of the largest solid tumor diameter (black arrow) (39 mm). **C** Both solid and cystic components were included in the calculation of total tumor volume (TTV) (blue area), which was 43.3 cm^3^. **D** Only solid tumor part was measured in STV (red area), which was 36.1 cm^3^

**Fig. 2:**
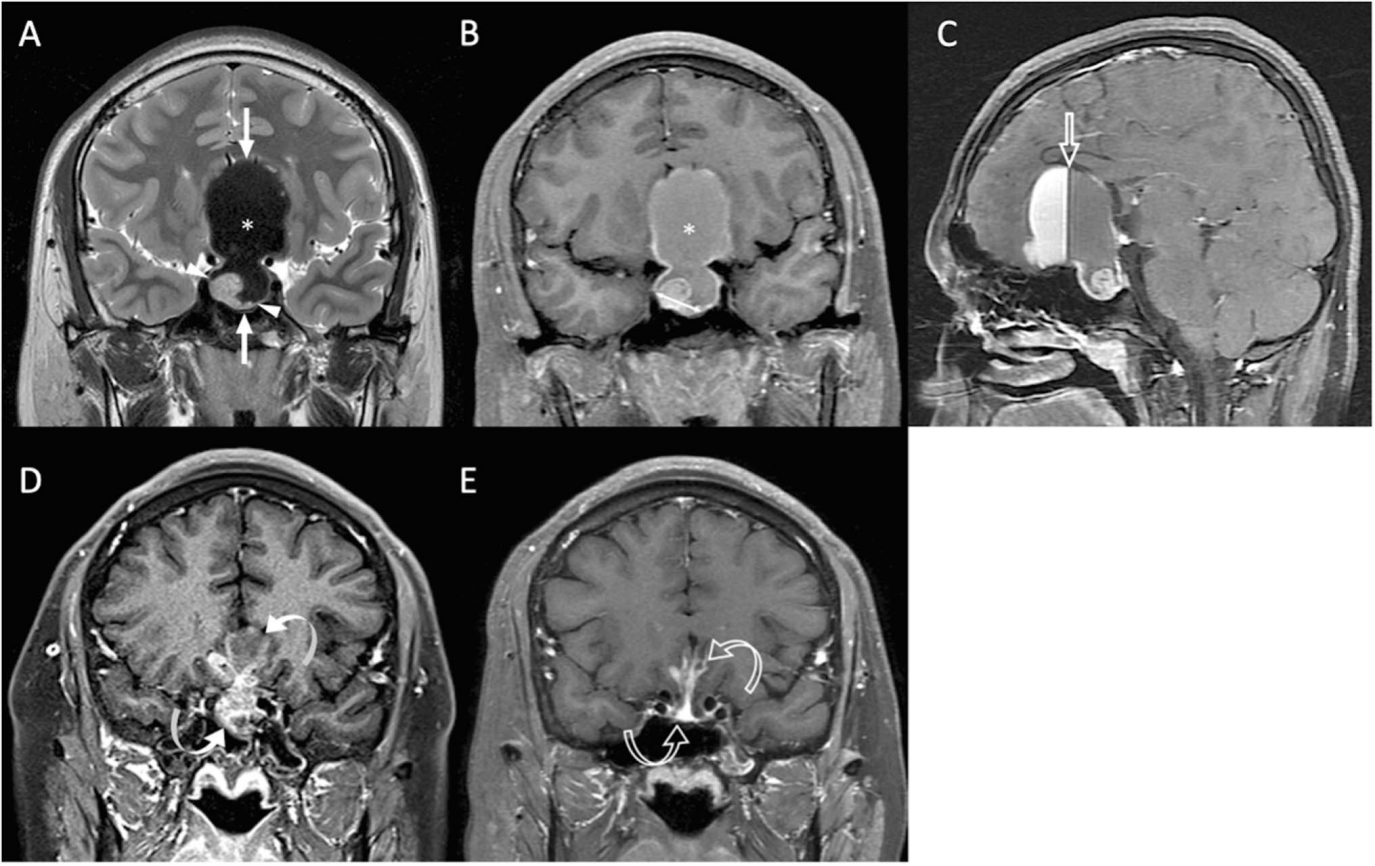
An 18-year-old male patient with blurred vision and pathologically confirmed NFPA. **A** Coronal T2WI (A) shows a gNFPA (> 40 mm) (white arrows) with upward suprasellar extension, causing compression of the optic chiasm and the third ventricle (cannot be seen). Intratumoral apoplexy (white star) and solid tumor part (arrowheads) were observed. **B** The STD (white double-headed arrow) measured on coronal CE T1WI was 15 mm, and the STV was 1.6 cm^3^. In contrast, the measured MTD and TTV were 61 mm and 43.3 cm^3^. **C** Sagittal CE T1WI showed intratumoral fluid–fluid level (open arrow) due to apoplexy. **D** Subtotal tumor resection via transsphenoidal approach (TSA) was performed, and the residual tumor (curved arrows) was observed. **E** Gradual shrinkage of residual tumor (open curved arrows) without recurrence was observed until 71 months after surgery

**Fig. 3:**
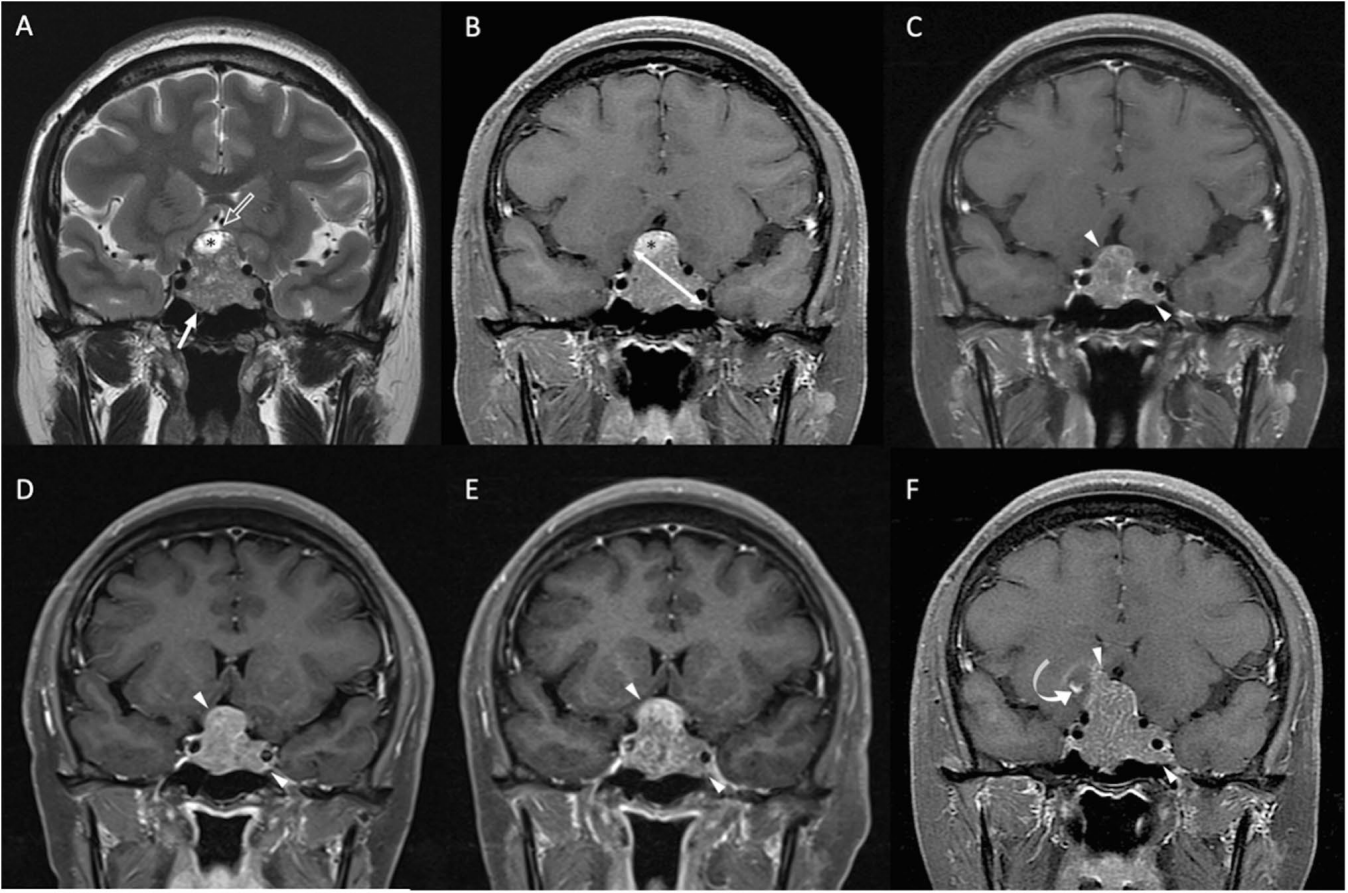
A 37-year-old female patient with blurred vision, amenorrhea, and pathologically confirmed NFPA. **A** Coronal T2WI shows a lNFPA (> 30 mm) tumor (white arrow) with upward suprasellar extension, causing compression of the optic chiasm and the third ventricle (open arrow indicates an area of the optic chiasm and third ventricle). Focal intratumoral cystic change (black star) was observed. **B** The STD (white double-headed arrow) measured on coronal CE T1WI was 29 mm, and the STV was 8.8 cm^3^. **C** Subtotal tumor resection via TSA was performed, and the diameter of the residual tumor (arrowheads) was 28 mm. **D**, **E**, **F** Progressive enlargement of the residual tumor (arrowheads) was observed at **D** 6 months (diameter of 31 mm), **E** 13 months (diameter of 36 mm), and **F** 56 months (diameter of 43 mm) after surgical resection. **F** Focal apoplexy (curved arrow) was also observed in the recurrent tumor (arrowheads)

Measurements of both tumor diameter and volume were obtained on coronal CE T1WI (Fig. 1) by using the freehand region of interest (ROI) tool on the Picture Archiving and Communication System (PACS) (INFINITT PACS; INFINITT Healthcare, Seoul, Republic of Korea) workstations. The preoperative maximal tumor diameter (MTD) is determined on coronal CE T1WI, and the STD is obtained by the only measurement of the solid tumor part. Preoperative total tumor volume (TTV) and postoperative residual volume (RV) are determined by manually calculating whole tumor areas in each coronal CE T1WI slice, and then compiling the volumes in the z-dimension using a semiautomated PACS measurement tool. The preoperative STV is obtained by the only measurement of solid tumor part without the involvement of intratumoral apoplexy, necrosis, or cystic change, which can be identified on coronal T2WI and CE T1WI (Figs. 1–3).

### Statistical analysis

Statistical analyses were performed using SPSS for Windows (V.24.0, IBM, Chicago, IL, USA). For evaluating clinical parameters and MR imaging features, chi-square (or Fisher exact test) and Mann–Whitney *U* tests are performed for categorical and continuous data respectively. The receiver operating characteristic (ROC) curve of STD, MTD, STV, and TTV for prediction of P/R was performed, and sensitivity, specificity, area under ROC (AUC), and optimal cutoff value were obtained. Further, Kaplan–Meier analyses based on cutoff values of STD and STV were used to evaluate the progression/recurrence-free survival (PFS), and the log-rank test was used to assess the significance. Cox proportional hazard regression model with univariate and multivariate analysis was performed to determine independent factors of P/R. Variables with a *p* value < 0.05 in univariate analysis were brought forward to the multivariate analysis. For all statistical analyses, *p* values < 0.05 were considered statistically significant.

The inter-observer reliabilities in the categorical and continuous data were determined using the Cohen *k* coefficient and intraclass correlation coefficient (ICC), respectively. The Cohen *k* coefficient and ICC were interpreted using the methods described by Landis et al. [22]. Both Cohen *k* coefficient and ICC with values between 0.8 and 1 were obtained, indicating almost perfect agreement. Due to almost perfect reproducibility in the ICC, the subsequent statistical evaluation of continuous data was performed using the mean value calculated from both readers.

## Results

### Clinical data and MRI features

The clinical data and MRI findings are summarized in Table 1. P/R is diagnosed in 23 (23/34, 67.6%) patients. Large preoperative STD and STV are more frequently observed in the P/R group (*p* < 0.05) (Figs. 2 and 3). In univariate Cox proportional hazards analysis (Table 2), significantly larger STD/STV and lesser EOR were observed in the P/R group (*p* < 0.05). Further, large STV is a risk factor for P/R (*p* < 0.05) with a hazard ratio of 30.79 in multivariate analysis (Table 2).

**Table 1:** Clinical and MRI characteristics of large and giant NFPA with and without P/R

	P/R (*n* = 23)	Non-P/R (*n* = 11)	***p***
Number	23	11	
Gender			0.262
Male	16 (69.6%)	5 (45.5%)	
Female	7 (30.4%)	6 (54.5%)	
Age	53 (45.5, 60.5)	45 (20.5, 69.5)	0.143
Clinical symptoms			
Visual disturbance	22 (95.7%)	10 (90.9%)	1
Headache	9 (39.1%)	5 (45.5%)	1
Symptoms of sex hormones	3 (13%)	1 (9.1%)	1
Incidental	1 (4.3%)	0	1
Hypopituitarism			0.925
Single	5 (21.7%)	2 (18.2%)	
Multiple	5 (21.7%)	2 (18.2%)	
Hyperprolactinemia (< 100 ng/mL)	8 (34.8%)	5 (45.5%)	0.709
Cavernous sinus invasion (Knosp grade 3–4)	11 (47.8%)	4 (36.4%)	0.715
Extrasellar extension (Hardy’s grade 3–4)	12 (52.2%)	5 (45.5%)	0.714
Compression of optic chiasm	22 (95.7%)	11 (100%)	1
Compression of 3^rd^ ventricle	22 (95.7%)	9 (81.8%)	0.239
Hydrocephalus	2 (8.7%)	1 (9.1%)	1
Apoplexy or cystic change	11 (47.8%)	9 (81.8%)	0.076
Successful chiasmatic decompression	7 (30.4%)	7 (63.6%)	0.135
Gross-total resection (GTR)	0	2 (18.2%)	0.098
Preoperative tumor size			
Maximal tumor diameter (MTD) (mm)	38 (33.5, 42.5)	36 (28.5, 43.5)	0.326
Total tumor volume (TTV) (cm^3^)	14.1 (7.5, 20.7)	10.5 (4.6, 16.4)	0.344
Solid tumor diameter (STD) (mm)	36 (29.5, 42.5)	21 (10, 32)	0.009*
Solid tumor volume (STV) (cm^3^)	12.7 (5.7, 19.7)	6.3 (3.7, 16.8)	0.007*
Residual volume (cm^3^)	5.9 (0.5, 11.3)	3.9 (1.0, 6.9)	0.082
Extent of resection (EOR) (%)	47 (27, 67)	81 (36, 89)	0.053
Follow up time (month)	41 (20, 62)	29 (12, 46)	0.098

Continuous variables were presented as median and interquartile range (IQR)

^*^Statistical difference (*p* < 0.05) in chi-square or Mann–Whitney *U* tests

**Table 2:** Cox proportional hazards analysis for P/R

	Univariate analysis		Multivariate analysis	
	HR (95% CI) for P/R (*n* = 34)	***p***	HR (95% CI) for P/R (*n* = 34)	*p*
Sex (fraction male)	2.74 (0.62, 12.08)	0.182		
Age (years)	1.04 (0.99, 1.09)	0.476		
Hyperprolactinemia	0.64 (0.15, 2.77)	0.550		
Cavernous sinus invasion (Knosp grade 3–4)	1.60 (0.37, 7.02)	0.530		
Extrasellar extension (Hardy’s grade 3–4)	1.31 (0.31, 5.53)	0.714		
Compression of 3rd ventricle	4.89 (0.39, 60.92)	0.218		
Apoplexy or cystic change	0.20 (0.04, 1.16)	0.073		
Successful chiasmatic decompression	0.25 (0.06, 1.14)	0.073		
Maximal tumor diameter (mm)	1.30 (0.55, 3.07)	0.545		
Total tumor volume (cm^3^)	1.02 (0.96, 1.07)	0.584		
STD > 26 mm (cutoff value)	38.5 (3.67, 403.93)	0.002*		
STV > 7.6 cm^3^ (cutoff value)	28 (3.92, 199.94)	0.001*	30.79 (2.25, 420.76)	0.01*
Residual volume (cm^3^)	1.04 (0.97, 1.13)	0.276		
Extent of resection (EOR) (%)	0.97 (0.94, 1.00)	0.041*	1.00 (0.96, 1.05)	0.866
Follow up time (month)	1.02 (0.99, 1.05)	0.212		

^*^Statistical difference (*p* < 0.05) in Cox proportional hazard regression analysis

### ROC and Kaplan–Meier analyses in solid tumor size

**Fig. 4:**
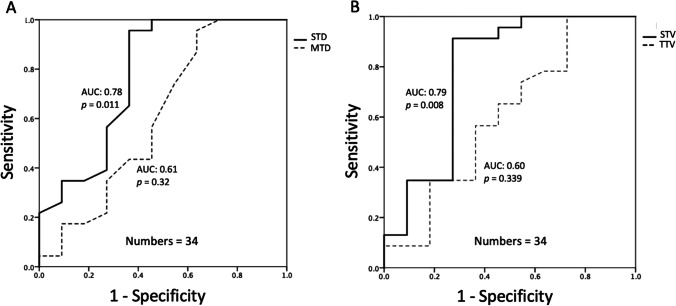
Receiver operating characteristic (ROC) curves of preoperative STD, MTD, STV, and TTV for prediction of P/R in lNFPA and gNFPA. AUC values of 0.78 and 0.79 with cutoff points of 26 mm and 7.6 cm^3^ were observed in STD (**A**) and STV (**B**), respectively

**Fig. 5:**
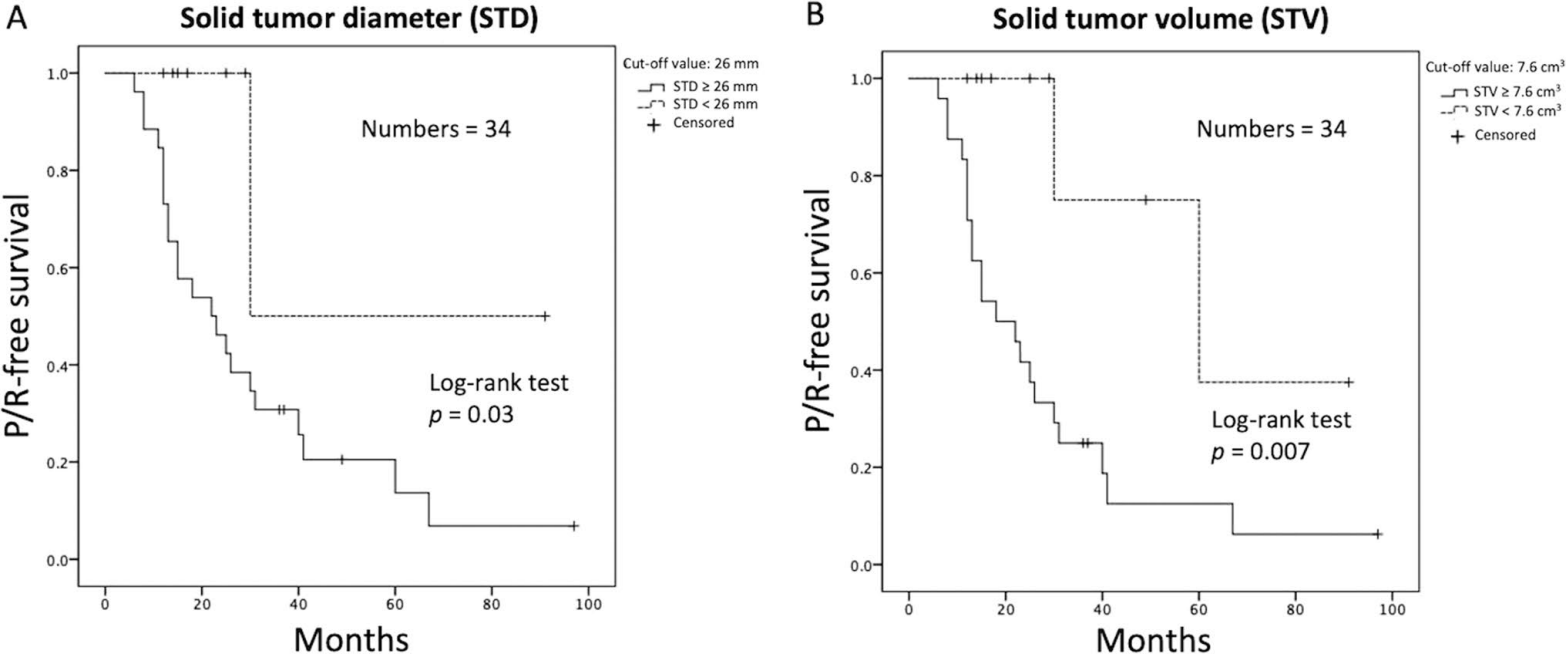
Kaplan–Meier survival curves showing significantly different (*p* < 0.05) progression/recurrence (P/R)-free survival based on cutoff points of preoperative STD (**A**) and STV (**B**) in lNFPA and gNFPA

The median follow-up duration for all patients was 47.6 months. In 23 patients with P/R, the median time to P/R is 25.2 months. The sensitivity, specificity, AUC, and optimal cutoff points of the STD and STV for differentiation between the P/R and non-P/R groups are summarized in Table 3. The cutoff points for the STD and STV ratio were 26 mm and 7.6 cm^3^, respectively. An AUC of 0.78, 0.61, 0.79, and 0.60 were obtained for the STD, MTD, STV, and TTV respectively (Fig. 4). When comparing the tumor P/R trends in Kaplan–Meier analysis, patients with larger STD (more than the cutoff value of 26 mm) and larger STV (more than the cutoff value of 7.6 cm^3^) exhibited shorter PFS (*p* < 0.05) (Fig. 5).

**Table 3:** ROC analysis of STD and STV for differentiating large and giant NFPA with and without P/R

(*n* = 34)	Sensitivity	Specificity	AUC	Cutoff value	*p*
Solid tumor diameter (STD)	0.96	0.64	0.78	26 mm	0.011*
Solid tumor volume (STV)	0.91	0.73	0.79	7.6 cm^3^	0.008*

^*^Statistical difference (*p* < 0.05) in ROC analysis

## Discussion

The purpose of this study was to analyze preoperative solid tumor size in predicting P/R of lNFPA and gNFPA after resection. The results showed that large STD and STV are significantly associated with P/R. Although the risk factors for the recurrence of NFPA have been previously reported, the present results are the first to offer quantitative cutoff points of preoperative solid tumor size for the prediction of P/R in lNFPA and gNFPA.

Although most NFPA are benign pituitary adenomas, 12–46% of them may show early P/R within 5 years after surgical resection [6, 7, 40]. According to 2017 WHO classification [27], tumor invasion and tumor proliferation index (Ki-67 and mitotic count) are associated with aggressive clinical behavior in NFPA. However, the definition of tumor invasion for NFPA was not clear in the WHO criteria and hence cannot be estimated if no corresponding information from MRI studies is considered [40, 44]. In the past 30 years, several meta-analyses consistently reported higher recurrence rates in NFPA than in secreting PA [6, 33, 37, 40]. Roelfsema et al. [40] showed that postoperative hormone concentration is an important predictor for P/R in functioning PA; however, no specific predictor is identified for NFPA. In contrast to functioning PA, for which biochemical markers often suggest tumor recurrence before the visible tumor is detected on imaging, tumor remission or recurrence in NFPA is mainly determined by MR imaging [29].

Some MR imaging features for the prediction of EOR and clinical outcomes in lNFPA and gNFPA had been reported [17, 18, 36]. Invasion of the cavernous sinus, maximum tumor diameter, and absence of tumor apoplexy were associated with an unfavorable surgical outcome in NFPA [28]. Invasion of the cavernous sinus is significantly associated with incomplete resection and residual tumor [2]. In contrast, more complete resection and less tumor recurrence could be achieved in NFPA with apoplexy [1]. This may explain why tumor recurrence is not significantly associated with the largest tumor diameter and total tumor volume, which are measured on both solid and apoplexy/cystic components of NFPA. RECIST is based on one-dimensional measurement of tumor size and is the gold standard for the evaluation of therapeutic response in solid tumors. However, a modified RECIST for hepatocellular carcinoma was developed by Lencioni et al. [26] based on the concept that a viable tumor should be defined as only intratumoral enhancing solid tumor part [3]. Lee et al. [24] reported that measurement of only solid tumor mass offers a better assessment of treatment response as compared with conventional RECIST in patients receiving targeted therapies for lung cancer. Similarly, association between solid tumor size and recurrence rate in lNFPA and gNFPA were observed in our study. On the other hand, low apparent diffusion coefficient value (diffusion restriction) is associated with recurrence in NFPA [21, 42]. Recently, quantitative MRI-based radiomics is also used for the evaluation of tumor behaviors in NFPA [46]. However, these parameters need to be measured on advanced MRI sequences and analyzed with complex statistical algorithms. The concept of simple and straight morphologic measurement focusing on solid tumor size for the prediction of clinical outcomes in NFPA is first mentioned in our study. The results offer quantitative, fast, and consistent measurement for neurosurgeons, radiologists, and clinical physicians in the evaluation of lNFPA and gNFPA.

EOR is a significant determining factor in the rate of recurrence in NFPA [2, 6, 29]. As the residual tumor due to incomplete surgical resection exists in most large and giant NFPA after surgery [18, 36], the issue of tumor recurrence is particularly important in this subgroup. Lee et al. [25] reported a recurrence rate of 8.2% and 58.3% in patients receiving GTR and STR, respectively. Maletkovic et al. [29] revealed that postoperative residual tumor confers a tenfold increased risk of recurrence in NFPA. Similarly, the association between EOR and P/R was observed in univariate regression analysis in our study. No statistical difference in the multivariate analysis may be explained by the small sample size and the association between EOR and solid tumor size.

It is known that postoperative adjuvant RT or stereotactic radiosurgery (SRS) is highly effective in preventing P/R in PA [23, 31]. Lee et al. [23] showed that empirical SRS was superior to progression-guided SRS for NFPA after subtotal resection. Although adjuvant RT and SRS may increase risks of radiation-induced complications such as hypopituitarism, neurocognitive dysfunction, cerebrovascular disease, and secondary brain tumors, the overall rate of serious complications is low [11, 14, 17, 32]. Progressive and irreversible hypopituitarism is the most commonly reported late complication, up to 20–30% at 5 years after treatment [17]. Since most NFPA are benign tumors, prediction of tumor recurrence offers clinically valuable information for treatment options. For patients with high possibilities of P/R, aggressive surgical resection combined with postoperative adjuvant RT and close MRI follow-up should be considered. In contrast, the surgery would aim to relieve clinical symptoms by decreasing tumor mass effects for patients with lower possibilities of tumor recurrence. Patients receiving adjuvant RT before P/R were excluded from our study because RT may affect the independent prediction of the preoperative MR imaging analysis for P/R.

The results of the current study propose cutoff values of solid tumor size for the preoperative prediction of P/R in NFPA. However, there are still several limitations in this study. Selection bias may exist due to its retrospective nature. As in other ROI-based studies, subjective freehand ROIs might influence the accuracy of the tumor size measurements. The small sample size may limit statistical power to detect potential associations between clinical or imaging parameters and P/R. Finally, there is a lack of complete histopathologic findings such as Ki-67 (MIB-1) and genomic signature for correlation in this retrospective study.

## Conclusions

lNFPA and gNFPA with larger solid tumor part were associated with higher possibilities of recurrence. The preoperative solid tumor diameter and volume for the prediction of P/R offer clinically useful information for the planning of NFPA treatment, including the extent of surgical resection, implementation of post-operative adjuvant RT, and the MR imaging follow-up strategy.

## Supplementary Information

Below is the link to the electronic supplementary material.

Supplementary file1 (DOCX 14 KB)

## Data Availability

Not applicable

## Data availability

The original contributions presented in the study are included in the article and supplementary material. Further inquiries can be directed to the corresponding author.
