# Correction to: Solid tumor size for prediction of recurrence in large and giant non-functioning pituitary adenomas

**DOI:** 10.1007/s10143-021-01680-5

**Published:** 2021-11-20

**Authors:** Ching-Chung Ko, Chin-Hong Chang, Tai-Yuan Chen, Sher-Wei Lim, Te-Chang Wu, Jeon-Hor Chen, Yu-Ting Kuo

**Affiliations:** 1 Department of Medical Imaging, Chi-Mei Medical Center, Tainan, Taiwan; 2 Department of Health and Nutrition, Chia Nan University of Pharmacy and Science, Tainan, Taiwan; 3 Department of Neurosurgery, Chi Mei Medical Center, Tainan, Taiwan; 4 Graduate Institute of Medical Sciences, Chang Jung Christian University, Tainan, Taiwan; 5 Department of Neurosurgery, Chi-Mei Medical Center, Chiali, Tainan, Taiwan; 6 Department of Nursing, Min-Hwei College of Health Care Management, Tainan, Taiwan; 7 Department of Radiological Sciences, University of California, Irvine, CA USA; 8 Department of Radiology, E-DA Hospital, I-Shou University, Kaohsiung, Taiwan; 9 Department of Medical Imaging, Kaohsiung Medical University Hospital, Kaohsiung, Taiwan

**Correction to: Neurosurgical Review**

10.1007/s10143-021-01662-7

The article “Solid tumor size for prediction of recurrence in large and giant non-functioning pituitary adenomas”, written by Ching-Chung Ko, Chin-Hong Chang, Tai-Yuan Chen, Sher-Wei Lim, Te-Chang Wu, Jeon-Hor Chen, and Yu-Ting Kuo, was originally published electronically on the publisher’s internet portal on October 4, 2020 without open access. With the author(s)’ decision to opt for Open Choice, the copyright of the article changed on October 7, 2021 to © The Author(s) 2021 and the article is forthwith distributed under a Creative Commons Attribution 4.0 International License, which permits use, sharing, adaptation, distribution and reproduction in any medium or format, as long as you give appropriate credit to the original author(s) and the source, provide a link to the Creative Commons license, and indicate if changes were made. The images or other third party material in this article are included in the article's Creative Commons license, unless indicated otherwise in a credit line to the material. If material is not included in the article's Creative Commons license and your intended use is not permitted by statutory regulation or exceeds the permitted use, you will need to obtain permission directly from the copyright holder. To view a copy of this license, visit http://creativecommons.org/licenses/by/4.0/.

The original article has been corrected.

